# Coastal exposure and residents’ mental health in the affected areas by the 2011 Great East Japan Earthquake and Tsunami

**DOI:** 10.1038/s41598-021-96168-z

**Published:** 2021-08-18

**Authors:** Ai Tashiro, Mana Kogure, Shohei Nagata, Fumi Itabashi, Naho Tsuchiya, Atsushi Hozawa, Tomoki Nakaya

**Affiliations:** 1 Tokushima University Graduate School of Biomedical Sciences, 3-18-15, Kuramoto-cho, Tokushima City, Tokushima 770-8503 Japan; 2 Tohoku Medical Megabank Organization, Tohoku University, 2-1 Seiryo-machi, Aoba-ku, Sendai, Miyagi 980-8573 Japan; 3 Graduate School of Environmental Studies, Tohoku University, 468-1, Aoba, Aramaki, Aoba-ku, Sendai, Miyagi 980-8572 Japan

**Keywords:** Environmental social sciences, Natural hazards, Disease prevention, Public health, Quality of life

## Abstract

No previous study has ever explored the association between coastal exposure and the mental health of residents in a post-disaster context. Therefore, we aimed to confirm whether there was an association between sea visibility and coastal proximity and the mental health of coastal residents a devastating tsunami. We targeted 15 coastal municipalities located in the Miyagi Prefecture, and obtained data from a community-based cohort study. The baseline survey was initiated 2 years after the 2011 Great East Japan Earthquake and Tsunami and the secondary survey was initiated 6 years after the disaster. We applied multilevel mixed-effects models to the longitudinal data. Our outcome measure was the Kessler Psychological Distress Scale (K6) score. We assessed the data collected from 2,327 respondents on both surveys as of April 2018 for this ongoing cohort study. We found that neither sea visibility nor coastal proximity was significantly associated with the recovery of mental health after the disaster. However, we found a distinctive trend of mental health recovery in people who lived alone with a sea view, indicating that visibility of the sea had a negative effect on their mental health immediately after the GEJET, but that the negative effect was subsequently eliminated.

## Introduction

Every year, individuals and communities are affected by natural disasters, which can disrupt their mental health and well-being^1^. Besides the trauma of the disaster itself, the aftermath itself can affect their mental health^2^. Residents who live near the coast are situated in the most sensitive zones for natural disasters, such as tsunamis, typhoons, and hurricanes. As of 2017, about 40% of the world’s population lives within 100 km of coastal regions^3^. In the 2011 Great East Japan Earthquake and Tsunami (GEJET), the most affected areas were the coastal Iwate, Miyagi, and Fukushima Prefectures. 93% of the post-disaster related deaths in those areas (14,632 of the 15,673 deaths reported in 2012) were of coastal residents^4–6^.

Despite the threat of natural disasters affecting the health of coastal residents, various studies have shown the positive effects of being exposed to the sea, such as the improvement of physical/mental health and overall well-being in daily lives^7–11^. Seascapes have recently received increasing attention as people have come to appreciate sea views^12^. Previous studies have demonstrated the positive impacts of sea views and coastal proximity on residents’ mental health^12–14^. In particular, environments that feature a water body are associated with a higher preference for the environment, greater positive effects (with respect to stress, blood pressure, and immune system etc.), and higher perceived restorativeness^14^. Another study reported that living near the coast was associated with better mental health for urban adults, with this tendency particularly obvious for the lowest-earning households^9^. This indicates that sea-related environmental factors are more likely to affect the mental health of vulnerable socio-demographic populations.

However, no previous study has ever explored whether exposure to the sea prolongs the adverse psychological effects of a disaster or improves the residents’ mental health in a post-disaster context. Immediately after a disaster, sea view or proximity to the sea appears to be related to the low mental health of residents of coastal areas due to the trauma and the aftermath of the disaster. Nevertheless, a sea view and proximity to the sea may contribute to the improvement of mental health due to the proven impact of sea exposure on residents’ mental health. These possible relationships between sea exposure and residents’ mental health may vary based on different socio-demographic subgroups (positive effects may be prominent only for sociodemographically vulnerable populations, such as those with low income, low educational attainment, and living alone). Loneliness, in particular, is a major risk factor for several physical and mental diseases^15^. In a post-disaster context in the Tohoku region, social isolation and loneliness, especially among older people, are regarded as vulnerable socio-demographic factors with respect to mental distress^16,17^.

Thus, considering socio-demographic vulnerabilities, we aimed to explore the association between exposure to the sea (i.e., coastal proximity and visibility of the sea) and the changes in residents’ mental health in the aftermath of the GEJET. This is the first longitudinal cohort study to examine this relationship in a post-disaster context.

## Results

### Socio-demographic characteristics of study participants

Table 1 presents the characteristics of the respondents at the baseline (May 2013 to March 2016) and secondary surveys (May 2017 to April 2018). The period between the baseline and secondary surveys ranged from 1.9 to 4.9 years. At the baseline, male participants accounted for 36.4% (n = 846) of the respondents. The gender ratio (males per 100 females) was 57.1. The distribution of our sample age was inclined toward 60 years or older (baseline: n = 1,665, 71.6%; secondary: n = 1,829, 78.6%). At the baseline, the gender ratio of those aged 60 years or older (male/female) was 112.2. Regarding household size (number of family members living with), the proportion of single-member households (living alone) was 7.4% (n = 171), and the most common household size at baseline was 2 or 3 (n = 1,405, 60.3%). In this study, the percentage of respondents aged 60 and over living alone in 15 municipalities of the Miyagi prefecture (hereafter referred to as coastal Miyagi) was 5.9% at baseline and 6.5% at the secondary survey.

**Table 1:** Socio-demographic characteristics of study participants at baseline and secondary survey (n = 2,327).

	Baseline survey				Secondary survey			
	n	%	Mean	SD	n	%	Mean	SD
K6^a^	…	…	4.7	4.5	…	…	3.0	3.3
Sex								
Male	846	36.4	…	…	…	…	…	…
Female	1,481	63.6	…	…	…	…	…	…
Total	2,327	100.0	…	…	…	…	…	…
Age								
20–39 years	118	5.1	…	…	79	3.4	…	…
40–49 years	177	7.6	…	…	136	5.8	…	…
50–59 years	367	15.7	…	…	283	12.2	…	…
60–69 years	1,179	50.7	…	…	981	42.2	…	…
70 years or more	486	20.9	…	…	848	36.4	…	…
Total	2,327	100.0	…	…	2,327	100.0	…	…
Educational attainment								
9 years or less	252	10.8	…	…	…	…	…	…
10–12 years	1,292	55.5	…	…	…	…	…	…
13 years and more	739	31.8	…	…	…	…	…	…
Others	27	1.2	…	…	…	…	…	…
Missing	17	0.7	…	…	…	…	…	…
Total	2,327	100.0	…	…	…	…	…	…
Loss of family and/or close relative and/or close friends in the disaster								
No	586	25.2	…	…	…	…	…	…
Yes	1,245	53.5	…	…	…	…	…	…
Missing	496	21.3	…	…	…	…	…	…
Total	2,327	100.0	…	…	…	…	…	…
Household size (people)								
Alone	171	7.4	…	…	…	…	…	…
2 or 3	1,405	60.3	…	…	…	…	…	…
4 or more	710	30.5	…	…	…	…	…	…
Missing 3	41	1.8	…	…	…	…	…	…
Total	2,327	100.0	…	…	…	…	…	…
Employment status								
Not working	1,376	59.1	…	…	…	…	…	…
Working	930	40.0	…	…	…	…	…	…
Missing	21	0.9	…	…	…	…	…	…
Total	2,327	100.0	…	…	…	…	…	…
Housing damage								
No damage	476	20.5	…	…	…	…	…	…
Some damage	1,764	75.8	…	…	…	…	…	…
Missing	87	3.7	…	…	…	…	…	…
Total	2,327	100.0	…	…	…	…	…	…
Moving after baseline survey								
No	…	…	…	…	2,100	90.2	…	…
Yes	…	…	…	…	227	9.8	…	…
Total	…	…	…	…	2,327	100.0	…	…
Sea visibility								
No	1,180	50.7	…	…	1,180	50.7	…	…
Yes	1,147	49.3	…	…	1,147	49.3	…	…
Total	2,327	100.0	…	…	2,327	100.0	…	…
Proximity: closest distance to ocean (mean = 3.67 km)								
Near (3.67 km or less)	1,484	63.8	…	…	1,490	64.0	…	…
Far (over 3.67 km)	843	36.2	…	…	837	36.0	…	…

Mean represents averaged scores among the respondents. Empty cells at secondary survey due to time-invariant variables.

SD, standard deviation.

^**a**^Continuous variables.

Regarding education achievement, more than half of the respondents had under 13 years of education (n = 1,544, 66.3%). Among the respondents, 1,245 (53.5%) reported having lost family members, relatives, and/or friends in the disaster, while 1,764 (75.8%) reported that some form of damage to their primary residence. In addition, 227 (9.8%) moved to a different residence within the field of the study after the baseline survey. Over half of the respondents reported having a view of the sea, a proportion which did not differ significantly between the baseline and secondary surveys, nor was there a significant difference in the average proximity to the coastline between the baseline and secondary surveys (mean = 3.67 km, standard deviation [SD] = 3.2 km). The number of primary school areas was 88.

### Sea exposure and mental health

As presented in Model 1 (two-way interaction) and Model 2 (three-way interaction) in Table 2, multiple imputation models for multilevel mixed-effects indicated no significant association between sea exposure (sea visibility and proximity) and mental health in Model 1 and Model 2. The survey period showed an improvement in mental health from the baseline survey to the secondary survey (coefficient = − 1.32, 95% CI: − 1.58, − 1.06, and coefficient = − 1.58, 95% CI: − 1.97, − 1.19, respectively). Regarding socio-demographic characteristics, both baseline and secondary surveys showed that ages 60 years and above was associated with better mental health in Model 1 and Model 2, that having more than 10 years of education was significantly associated with better mental health, and that the loss of family and/or intimate persons in the disaster was significantly associated with worsened mental health.

**Table 2:** Multilevel regression adjusted coefficient and 95% confidence interval of individual attributes and coastal exposure (sea visibility and coastal proximity) from multiple imputation analysis for changes in mental health (Kessler Psychological Distress Scale score) in coastal municipalities, Miyagi, Japan, 2013–2018.

	Model 1 (two-way interaction)		Model 2 (three-way interaction)	
	Coef	95%CI	Coef	95%CI
Intercept	5.95***	[5.16, 6.74]	6.21***	[5.39, 7.04]
**Effects of environments**				
Sea visibility				
Non-visible	Ref	–	Ref	–
Visible	0.11	[− 0.23, 0.46]	− 0.29	[− 0.87, 0.30]
Proximity (mean of closest distance to the ocean = 3.67 km)				
Near (3.67 km or less)	− 0.06	[− 0.45, 0.33]	− 0.06	[− 0.45, 0.33]
Far (over 3.67 km)	Ref	–	Ref	–
**Two-way interaction effects of environments**				
Sea visibility × Survey period (reference = baseline)	0.07	[− 0.26, 0.39]	0.28	[− 0.27, 0.82]
Proximity × Survey period (reference = baseline)	− 0.31	[− 0.64, 0.02]	− 0.03	[− 0.65, 0.02]
Household number × Sea visibility (reference = non-visible)				
Alone × visible	–	–	1.28*	[0.02, 2.54]
2–3 × visible	–	–	0.50	[− 0.20, 1.20]
4 or more × visible	–	–	Ref	–
**Two-way interaction effect**				
Household number × Survey period (reference = baseline)				
Alone × secondary	–	–	1.10*	[0.24, 1.97]
2–3 × secondary	–	–	0.31	[− 0.13, 0.76]
4 or more × secondary	–	–	Ref	–
**Three-way interaction effects of visibility**				
Household number × Sea visibility × Survey period (reference = baseline)				
Alone × visible × secondary	–	–	− 1.43**	[− 2.65, − 0.21]
2–3 × visible × secondary	–	–	− 0.19	[− 0.84, 0.46]
4 or more × visible × secondary	–	–	Ref	–
**Control variables**				
Sex				
Female	Ref	–	Ref	–
Male	− 0.54***	[− 0.84, − 0.24]	− 0.55***	[− 0.85, − 0.25]
Age				
20–39 years	0.37	[− 0.32, 1.06]	0.35	[− 0.35, 1.04]
40–49 years	Ref	–	Ref	–
50–59 years	− 0.48	[− 0.98, 0.03]	− 0.46	[− 0.96, 0.05]
60–69 years	− 1.85***	[− 2.35, − 1.34]	− 1.83***	[− 2.34, − 1.33]
≤ 70 years	− 2.06***	[− 2.61, − 1.51]	− 2.05***	[− 2.60, − 1.51]
Educational attainment				
9 years and less	Ref	–	Ref	–
10–12 years	− 0.55*	[− 1.02, − 0.09]	− 0.54*	[− 1.01, − 0.08]
13 years and more	− 0.63*	[− 1.13, − 0.13]	− 0.63*	[− 1.13, − 0.12]
Others	− 0.82	[− 2.20, 0.57]	− 0.77	[− 2.15, 0.61]
Loss of family and/or close relative and/or close friends in the disaster				
No	Ref	–	Ref	–
Yes	0.64***	[0.26, 1.04]	0.64***	[0.26, 1.03]
Household size (people)				
Alone	1.07***	[0.49, 1.64]	0.24	[− 0.67, 1.15]
2 or 3	0.20	[− 0.15, 0.51]	− 0.16	[− 0.66, 0.33]
4 or more	Ref	–	Ref	–
Employment status				
Not working	Ref	–	Ref	–
Working	0.19	[− 0.11, 0.49]	0.19	[− 0.11, 0.49]
Housing damage				
No damage	Ref	–	Ref	–
Some damage	0.19	[− 0.17, 0.55]	0.19	[− 0.17, 0.54]
Moving after the baseline survey				
No	Ref	–	Ref	–
Yes	− 0.22	[− 0.71, 0.27]	− 0.22	[− 0.70, 0.27]
Survey period				
Baseline	Ref	–	Ref	–
Secondary	− 1.32***	[− 1.58, − 1.06]	− 1.58***	[− 1.97, − 1.19]
Random effects				
Individual-level	2.98	[2.86, 3.11]	2.98	[8.20, 9.67]
School district-level	0.23	[0.06, 0.83]	0.23	[0.06, 0.82]
Residual variance	2.49	[2.41, 2.56]	2.48	[2.41, 2.56]

Coef., adjusted coefficient; CI, confidence interval; Ref, reference.

*p < 0.05; **p < 0.01; ***p < 0.001.

In Model 1, regarding sea exposure effects, the main effects of having sea visibility and coastal proximity were not statistically significant (coefficient = 0.11, 95% CI: − 0.23, 0.46; coefficient = − 0.06, 95% CI: − 0.45, 0.33, respectively). The interaction term of visibility × survey period in Model 1 indicated that living with a sea view was not significantly associated with changes in mental health (coefficient = 0.07, 95% CI: − 0.26, 0.39). The interaction term of coastal proximity × survey period in Model 1 revealed that the K6 scores of those living near the sea (less than the mean) were also not significantly associated with changes in mental health (more than the mean) (coefficient = − 0.31, 95% CI: − 0.64, 0.02).

Model 2 (three-way interaction) is the model including the other significant interaction terms of the sea exposure variable and survey period. The two-way interaction term of household size × sea view visibility in Model 2 revealed that sea visibility was significantly negatively associated with mental health among participants living alone (coefficient = 1.28, 95% CI: 0.02, 2.54). Regarding three-way interaction term of sea view visibility, survey period, and household size, people living alone with a sea view showed signs of mental health recovery in the secondary survey (coefficient = − 1.43, 95% CI: − 2.65, − 0.21). To clarify how the change in the K6 score between the two survey periods occurred on average for the different household-size groups, we calculated the marginal effect of this interaction term of visibility.

**Figure 1:**
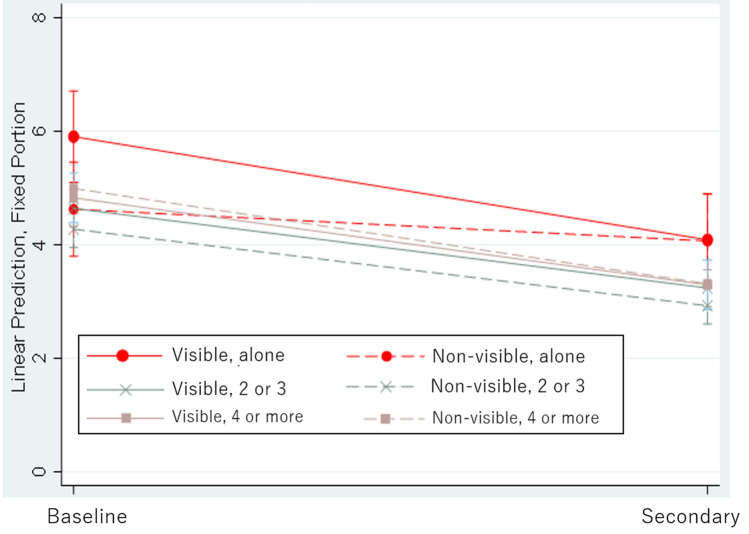
Predictive margins of Kessler Psychological Distress Scale score change by the interaction term of sea visibility and household size between the baseline and the secondary survey for living alone.

Figure 1 shows the results of the predicted margins of K6 in Model 2 (three-way interaction) between the two survey periods for those in the sample who lived alone. The predicted changes in the K6 score from the baseline survey to the secondary survey were − 1.91 (95% CI: − 2.69, − 1.13) for those living alone with a sea view and − 0.64 (95% CI: − 1.43, 0.15) for those living alone without a sea view respectively. The difference between the two visibility groups of − 1.27 (95% CI: − 2.38, − 1.67) indicated that the improvement in the K6 scores for those who lived alone with a sea view was significantly larger compared to those who did not (Supplement S4 (Table(B)).

We did not significantly improve the model performance by adding such additional two or three-way interaction terms with proximity (Supplement S4); therefore, the marginal effect of proximity was not shown.

## Discussion

The aim of this study was to examine whether exposure to the sea (i.e., coastal proximity and sea visibility) is associated with coastal residents’ mental health in a post-disaster context.

So far, most epidemiological studies on the aftermath of the GEJET linking disaster experiences with health outcomes have focused on individual characteristics or social support for elderly people^18–20^. The elderly and those who live alone are generally considered socially vulnerable because they may not have enough support. Elderly individuals with severe mental conditions may be reluctant to seek support from those around them, and their mental condition can worsen^20^.

In contrast, this study predominantly focused on the influence of blue space exposure on mental health outcomes, given previous findings on the effects of blue space on human health. For example, the sea has been shown to be a positive influence on human health^7–10^. Hooyberg et al. (2020) also suggested that the health benefits of living near the coast and the accessibility of these benefits for all socio-economic classes can help reduce social inequality and provide environmental justice^21^.

Inconsistent with several previous blue space studies^11–14^ that demonstrated the positive effect of coastal proximity on health outcomes, coastal proximity was not observed as an effective factor for improving mental health among individuals in disaster-affected areas. Dempsey et al. (2018) revealed that respondents with the highest level of sea visibility had lower depression scores^22^. They concluded that the increased visibility of coastal blue space, rather than its physical proximity, is what reduced the depression scores.

In contrast, our study indicated that sea visibility was a factor affecting mental health. However, the association between sea visibility and mental health was more complex for individuals living in disaster-affected areas. Unlike previous studies, this study did not find a significant association between sea exposure (sea visibility and coastal proximity) and K6 score at the baseline and the secondary survey. However, when we added the variable of living alone to the association of K6 score with sea exposure, we found a negative effect of sea visibility on K6 score at baseline. The coefficient for the interaction of living alone × sea visibility was significantly positive at the baseline survey (coefficient = 1.28; 95% CI, 0.02, 2.54).

We managed to clarify the effect of sea exposure on K6 score at the secondary survey after adding the following variable: (sea view visibility × living alone × survey period). The effect on K6 score was − 0.15 (1.28 + (− 1.43)). To compensate for this interpretation, we adjusted the values of other variables and conducted a post estimation for persons living alone, compared to those living with 2–3 members, and those with 4 members or more in Fig. 1.

Figure 1 indicated that those who lived alone with a view of the sea had worse K6 scores at baseline, but their K6 scores improved greatly in the secondary survey, and became on the same level as those who lived alone without a view of the sea.

While a study in the UK reported that living near the coast was more clearly associated with better mental health for low-income households^9^, we did not identify a similar trend for the interaction term of sea exposure and low education attainment in this study. There is less income-generating knowledge work in the rural areas than in urban areas, which could mean that the relationship between educational attainment and income is less clear. Another possible reason would be the cultural homogeneity of the rural coastal areas. In these areas, the seascape is familiar for residents, regardless of their educational background. It would depend on the local context as to what kind of people are likely to undergo spiritual recovery through their relationship with the sea. With regards to living alone, we assumed from our findings that the number of people living together, which is a living condition that is more susceptible to environmental changes as opposed to a fixed individual attribute (education attainment), is significantly associated with mental health and sea exposure.

In our study, the interaction variable of sea visibility and people living alone showed higher K6 score than those who lived with someone. This finding may indicate that sea exposure itself is a negative factor for the mental health of people living alone at the baseline. This can be explained by the fact that at the time of this study, a large proportion of people were still traumatized by the GEJET.

However, living near the sea might improve residents’ mental health outcomes, if we highlight their distinctive improvement in mental health from the baseline to the second survey. The analysis results indicated that the interaction term of living alone × sea visibility × secondary period was significantly associated with a lower level of psychological distress in this study. These findings help us to understand the backgrounds of residents in disaster-affected areas. Historically, most residents in these disaster-affected areas tend to have a strong emotional attachment to the sea^23^. Even when living away from it, a sea view may bring back descriptive memories, such as those of fishing, diving, paddling, and sitting on a beach. Such positive memories might contribute to their ability to overcome disaster-related trauma over time.

For those living alone, a lack of support from housemates may increase K6 scores if they don’t have a sea view. Alternatively, as shown in Fig. 1, the tendency of high K6 scores for them was not present in the secondary survey. In fact, the mental health of individuals living alone with a sea view was slightly better than those without a sea view in the secondary survey, even though the difference is statistically insignificant. Instead of family support, a potential seascape attachment could have helped in the mental health recovery for those living alone.

In conclusion, we found a distinctive trend of mental health recovery in people who lived alone with a sea view, indicating that visibility of the sea had a negative effect on their mental health immediately after the GEJET, but that the negative effect was subsequently eliminated. Considering the previous studies indicating the positive effect of sea visibility on mental health and the local context of the residents’ strong seascape attachment, it is possible that visibility of the sea had a positive effect on the mental health recovery of the people living alone. However, this effect needs to be further clarified in follow-up studies.

A major strength of this study is its scale. This study is the first large longitudinal cohort study to explore the effects of both sea visibility and coastal proximity on mental health in a post-disaster context in disaster-affected areas. This study makes a unique contribution to the literature regarding environmental factors and post-disaster development. After the 1995 Kobe earthquake, there was a problem where relocation sites for the disaster victims were decided without considering their original living environments^24^. Aldrich (2011) suggested that even though social capital was crucial in disaster recovery, it decreased among victims who were relocated to temporary housing due to housing allocation by random lottery^24^. Most of the victims had no choice in where they were relocated after the disaster. While we believe that policymakers should have paid more attention to the positive effect of environmental characteristics back then, this study provides empirical evidence supporting our claims by showing how environmental factors (sea visibility) can help these victims recover much faster.

We note a limitation in the lack of epidemiological representativeness. As shown in the Result section, compared to the Miyagi Prefectural Statistics of regional population^25,26^, the demographic data indicate that participants were older and more likely to be women than men, which could be considered a bias issue. More importantly, the data analyzed were from the middle period of the secondary survey. There might be a possibility that they included respondents whose mental health had recovered more quickly compared to those who did not complete the secondary survey until April 2018. Regarding our interpretation of the participants living alone with a sea view, even though their K6 score was better at the secondary survey compared to persons living alone without a sea view, the change was so small that a follow-up survey is needed to confirm the effect.

Furthermore, the data sample has significant differences in attrition rates in terms of age, education attainment, housing damage, employment status, bereavement, and household size (Supplement S1). In this sense, we note a limitation on the lack of epidemiological representativeness. The data sample we used was a relatively early, highly motivated population. This may have resulted in selection bias. However, there were no differences in population distribution of K6 score or sea exposure (sea visibility and coastal proximity). Thus, we believed our data sample was sufficient for main purpose of this study. The potential bias applied to both respondents who lived near the sea (lived with sea view) and those who did not and hence may also not have affected this study’s main findings.

## Methods

### Study design and participants

**Figure 2:**
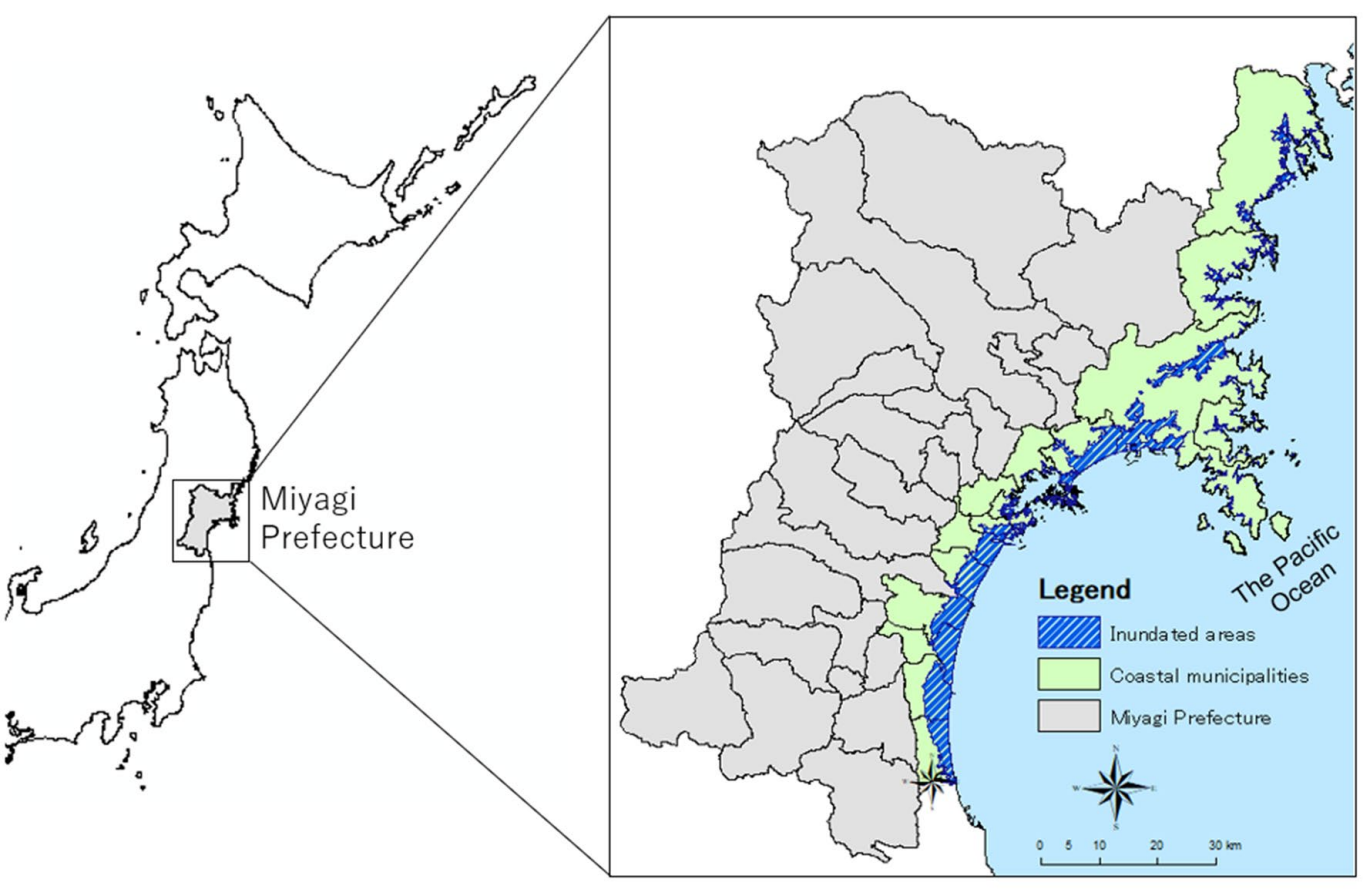
Study area (15 coastal municipalities) in Miyagi Prefecture, Japan. The map was created by authors using ArcGIS Pro 2.4 (https://www.esrij.com/products/arcgis-desktop/environments/arcgis-pro/).

This study was part of the Tohoku Medical Megabank Project Community-Based Cohort Study (TMM CommCohort Study)^27^. The baseline survey was conducted between 2013 and 2016 in Miyagi and Iwate Prefectures in Japan. This study employed data of individuals who lived in 15 coastal municipalities within Miyagi Prefecture (total population: approximately 0.94 million)^5^, located roughly 80 km west of the epicenter of the GEJET. This prefecture suffered severe and widespread damage, including 9,384 deaths and the inundation of 5% ﻿(32,700 ha)^5,28^ of land area by seawater (Fig. 2).

The TMM CommCohort Study^27^ mainly adopted two ways of recruiting for the baseline survey—one, they recruited people who visited specific health check-up sites and two, they recruited people who came for detailed health check-ups in assessment center-based sites in Miyagi Prefecture. We visited the specific health check-up sites and requested participants to join our survey. Participants living in Miyagi Prefecture were eligible for screening provided by their insurance company. Participants who took the health check-up in a municipality were mainly covered members of the National Health Insurance. These check-ups focused on visceral fat obesity with risk factors of cardiovascular disease, hypertension, diabetes, dyslipidemia, and metabolic syndrome^28^. The participation rate of the baseline survey was 70% (87,865 people gave their informed consent to join the study)^28^. Details of this information was provided in the previous study^28^. Informed consent was obtained at the time of the survey, or an informed consent form was mailed to the participants. All participants gave written informed consent for study inclusion.

The assessment centers were located in seven locations in Miyagi Prefecture, and participation in the survey was voluntary^28^. To avoid potential biases, this study only used data from participants recruited at health check-up sites because participants who provided informed consent at health check-up sites had different health consciousness compared to those who voluntarily joined the survey. This study was approved by the Tohoku Medical Megabank Organization Institutional Review Board (2019-4-065 and 2019-4-032).

The details of participant selection were as follows. In the baseline survey, 40,433 participants were recruited from specific health check-up sites. Of these, 37,222 participants completed questionnaire sheets during the baseline survey. We geocoded the data using their residential addresses at the baseline survey. Of these, we excluded 23,538 individuals who lived in inland municipalities and further excluded 400 participants who provided incomplete or no data on the K6 questionnaire at baseline. The baseline data included 13,239 participants who both lived in coastal municipalities and had completed the K6 questionnaire.

**Figure 3:**
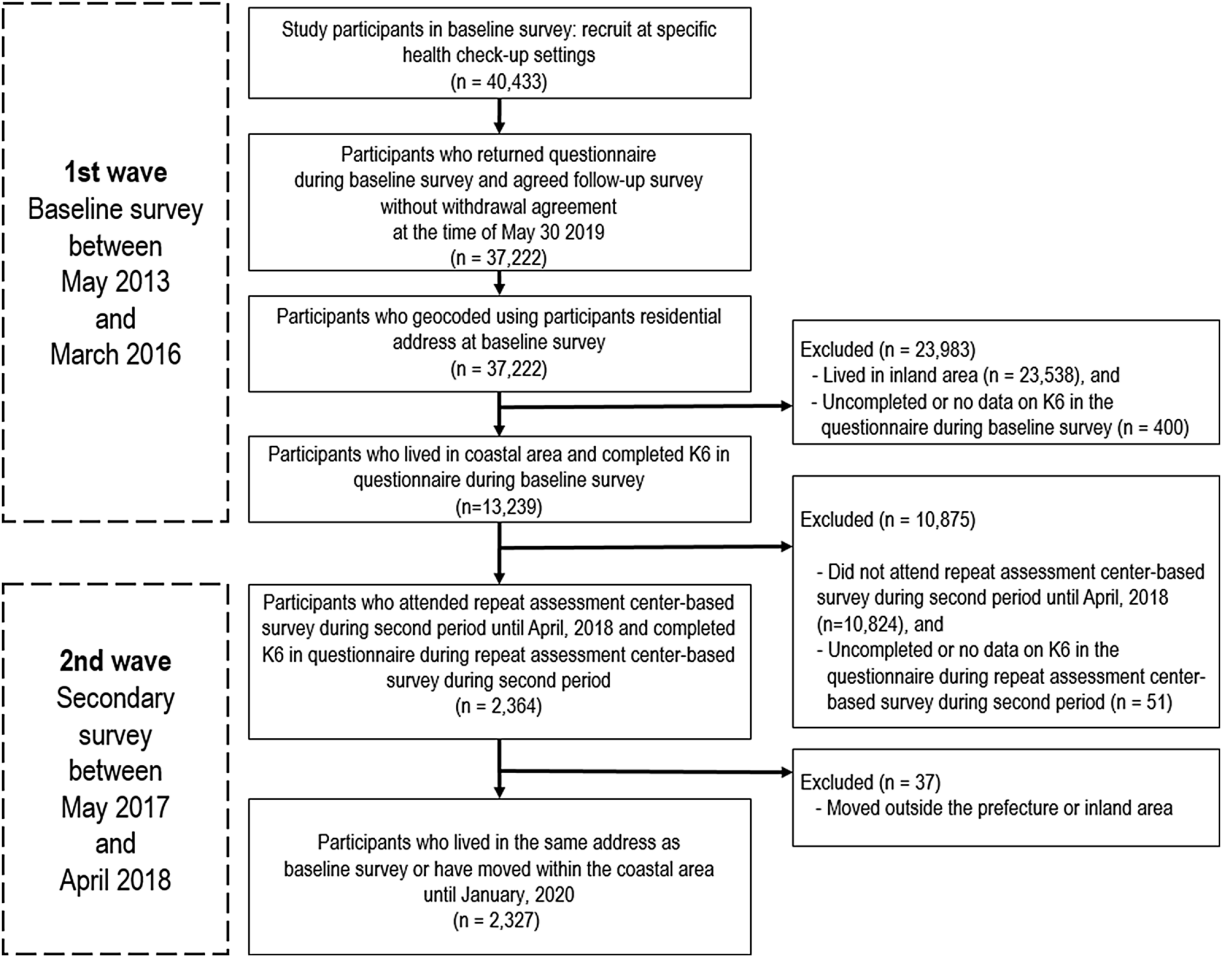
Flow chart for the analytical panel sample (n = 2,327) from 15 coastal municipalities, Miyagi, Japan, 2013–2018.

In the secondary survey, 10,824 participants did not attend a repeat assessment center-based survey during the second study period. A total of 2,415 respondents participated during the second wave period. Of these, 51 provided incomplete or no data on the K6 questionnaire. A further 37 respondents moved outside the prefecture or to an inland area during the secondary survey period. In total, the data of 2,327 respondents were used for the longitudinal data in this study (Fig. 3).

### Geographical analysis

The participants’ addresses were geocoded using the address geocoding function of ArcGIS (Esri Japan Inc., Tokyo, Japan). The percentages of matched complete addresses were 71% and 75% for the baseline and secondary samples respectively, and those matched more granularly at the residential block-level (*banchi*) were 23% and 19%, respectively. The rest of the addresses for both surveys were matched at the small neighborhood unit (*cho-aza*). We considered this level of geocoded addresses suitable to be used for the calculations of sea visibility and coastal proximity from the place of living.

### Residential coastal proximity and visibility

Coastal proximity was calculated by measuring the distance between the participants’ geocoded places of living and the nearest coastline as the crow flies using the spatial analyst function, Near in ArcMap 10.6.

To assess sea view visibility from the participants’ living place, this study followed the procedure of a previous seascape study in Japan^29^. This study generated a 200 m grid-point layer with a 3 km buffer from the coastline to the sea, established the grid points as observation points at sea level (0 m), and analyzed the raster 10 m grid’s visibility cells from these points.

For land elevation, we used the Fundamental Geospatial Data (Digital Elevation Model [DEM]) 10 m Mesh (Elevation) of the coastal land of Miyagi Prefecture, which was created by the Geospatial Information Authority (GIA) of Japan (which is part of the Ministry of Land, Infrastructure, Transport, and Tourism), as altitude data^30^.

**Figure 4:**
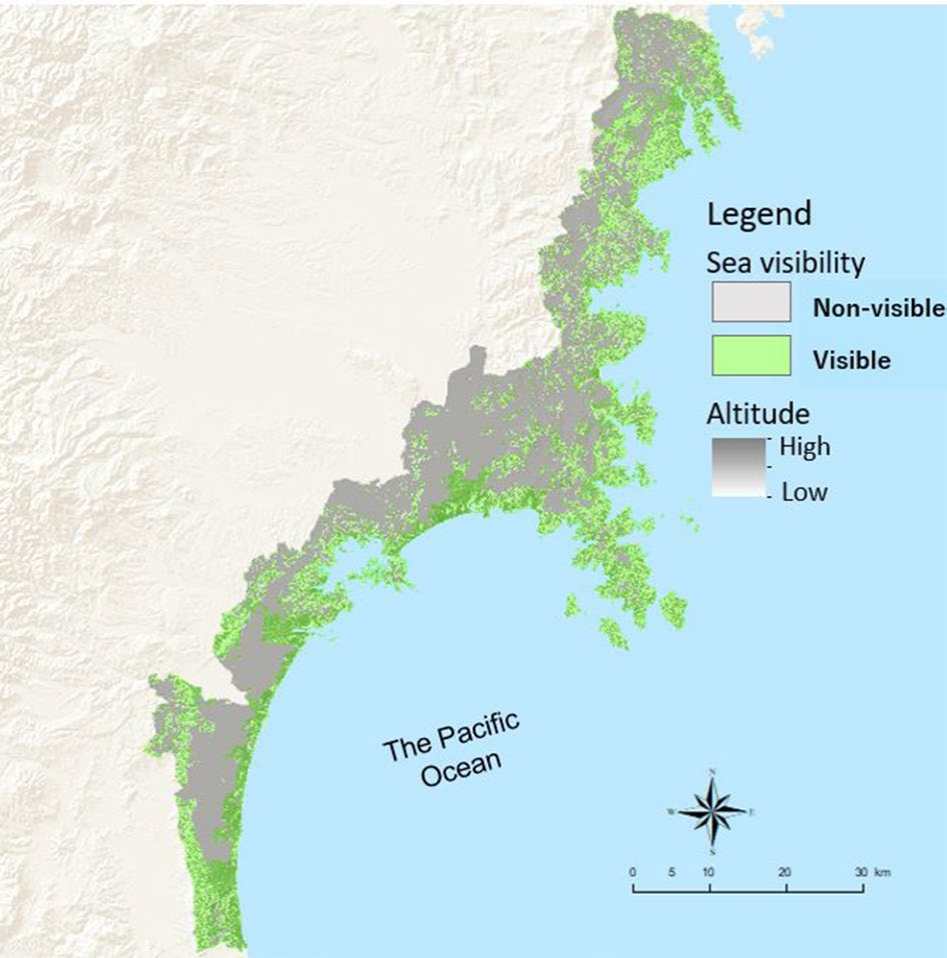
Sea visibility in the study site by viewshed analysis. The map was created by authors using ArcGIS Pro 2.4 (https://www.esrij.com/products/arcgis-desktop/environments/arcgis-pro/).

The average elevation in a 10 m × 10 m grid was defined as an attribution of the grid by the GIA. Considering the Japanese average height (1.65 m) and eye position (average height × 0.9 m)^31^, we added 150 cm to the altitude from the surface of the earth in the original DEM to establish the vertical viewing points. The visibility assessment analysis was conducted using the Viewshed 2 function in ArcGIS Pro 2.4. (https://www.esrij.com/products/arcgis-desktop/environments/arcgis-pro/). Figure 4 shows the results of the viewshed analysis of sea visibility in the study site. It should be noted that we did not assess the height of the respondents’ houses and buildings in the visibility analysis.

### Outcome variable

Our outcome variable was K6 score^32^, which measures the mental health of participants. The K6 consists of a six-item battery that asks how frequently participants have experienced symptoms of psychological distress in the past 30 days.

The response for each item ranges from 0 (none of the time) to 4 (all of the time) with the total score ranging from 0 to 24^33^. A higher score indicates a greater tendency toward mental illness. We used the score of the validated Japanese version of the K6 questionnaire as a continuous variable^34^.

### Explanatory variables and covariates

Our primary explanatory variables were sea visibility and coastal proximity. Based on the results of the viewshed analysis in ArcGIS Pro 2.4, if a participant’s place of living had a view of the sea, we categorized it as visible, and as non-visible if it did not. The value of the coastal proximity was the crow-fly distance from the participants’ place of living to the closest coastline (in km). The distance was dichotomized at the mean of 3.67 km.

We selected the following as time-variant confounding variables: age, employment status, change in housing after the baseline survey, sea visibility, and closest distance to the sea. The time-invariant characteristics were sex, education attainment, household size (number of people), loss of family and/or other loved ones and housing damage by the GEJET. While the education attainment did not change in the study sample, more than half of records for household size at the secondary survey was missing. As such, the author treated household size as a time-invariant variable to avoid the ambiguity caused by the missing information.

### Statistical analysis

We used multiple imputation for multilevel mixed-effects models with missing data to analyze the longitudinal data^35,36^. The model assumes that the individual observations are grouped in some way because of the design of the data. We created a three-level structure in the multilevel model, with repeated observations over time (Level 1) nested within individual changes (Level 2) and within coastal primary school areas (n = 88) (Level 3).

The model predicts the set of respondents’ K6 scores at the baseline and secondary survey as the dependent variable. Model 1 has covariates and the two-way interaction term of sea view visibility and survey period, as well as the two-way interaction term of coastal proximity and survey period (baseline and secondary surveys). While the main effects of sea exposure variables indicate whether sea exposure is associated with better/worse mental health at baseline, the coefficient of interaction term indicates whether these variables buffer the disaster’s effect on the K6 scores (i.e., improved mental health) from the baseline to the secondary survey.

To consider the effect of sea exposure on socially vulnerable groups, we applied three-way interaction terms among sea exposure (visibility), survey period, and covariates of respondents’ vulnerability characteristics in Model 2, namely: housing damaged by the GEJET, loss of important person(s) by the GEJET, low educational attainment, and living alone. In this model, we examined every possible three-way interaction term and retained the statistically significant variables, particularly to inspect possible differences of the associations between sea exposure and mental health among different socio-demographic subgroups.

The data was analyzed using Stata version 15.0 (StataCorp LP, College Station, TX, USA) and the level of significance was set *p* value for < 0.05.

### Ethical considerations

The survey protocol was approved by the human subjects’ committee of the Tohoku Medical Megabank Organization Institution, Tohoku University (approval no. 2019–4-065 and 2019-4-032). Informed consent was obtained from all participants at the time of data collection. Voluntary participation and right to withdraw at any time were assured. All methods were carried out in accordance with the applicable guidelines and regulations for the use and analysis of the Tohoku Medical Megabank Project Community-Based Cohort Study (TMM CommCohort Study), managed by the Tohoku Medical Megabank Organization, Tohoku University. This study conformed to the principles of the Declaration of Helsinki.

## Supplementary Information

Supplementary Information.

## Data Availability

Since the data with geographical information of this survey contains personal information, the data itself could not be accessed due to the project contract for privacy setting.
