# Supplementary material for: Coastal exposure and residents’ mental health in the affected areas by the 2011 Great East Japan Earthquake and Tsunami
Source: Sci Rep. 2021 Aug 18;11:16751. doi: 10.1038/s41598-021-96168-z (PMC8373874; doi:10.1038/s41598-021-96168-z)
Supplement: Supplementary file 1 — Supplementary Information. [file 41598_2021_96168_MOESM1_ESM.docx]

**Supplementary Information**

Coastal exposure and residents’ mental health in the affected areas by the 2011 Great East Japan Earthquake and Tsunami

Ai Tashiro^1^, Mana Kogure^2^, Shohei Nagata^3^, Fumi Itabashi^2^, Naho Tsuchiya^2^, Atsushi Hozawa^2^, Tomoki Nakaya^3^*

^1^Tokushima University Graduate School of Biomedical Sciences, 3-18-15, Kuramoto-cho, Tokushima City, Tokushima, 770-8503, Japan.

^2^Tohoku Medical Megabank Organization, Tohoku University, 2-1 Seiryo-machi, Aoba-ku, Sendai, Miyagi 980-8573, Japan

^3^Graduate School of Environmental Studies, Tohoku University, 468-1, Aoba, Aramaki, Aoba-ku, Sendai, Miyagi, 980-8572, Japan

*Correspondence to: Tomoki Nakaya (tomoki.nakaya.c8@tohoku.ac.jp)

**Contents**

**S1 Results of comparing the sample data of baseline between those who did not attend the secondary survey and those who joined the secondary survey until April 2018. The difference between the two populations was tested (χ2 and t-test).**

*p<0.05, **p<0.01, ***p<0.001.

|  | **Baseline (N = 13,239)** | | | | | | | | | |
| --- | --- | --- | --- | --- | --- | --- | --- | --- | --- | --- |
|  | **Group did not attend by April 2018** | | | | **Group attend by April 2018** | | | | | ***P-value*** |
|  | **Total n = 10,912** | | | | **Total n = 2,327** | | | | |  |
|  | **N** | **Mean**  **± SD** | **95% CI** | **％** | **N** | **Mean**  **± SD** | **95% CI** | **％** | **Proportion** |  |
| **K6 score** | 10,912 | 4.70 (±4.52) | (4.61, 4.78) | 100.0 | 2,327 | 4.67 (±4.51) | (4.49, 4.85) | 100.0 |  | 0.77 |
| 0-4 | 6,347 |  |  | 58.2 | 1,364 |  |  | 58.6 | 17.7 | 0.47 |
| 5-8 | 2,632 |  |  | 24.1 | 571 |  |  | 24.5 | 17.8 |  |
| 9-12 | 1,233 |  |  | 11.3 | 237 |  |  | 10.2 | 16.1 |  |
| 13-24 | 700 |  |  | 6.4 | 155 |  |  | 6.7 | 18.1 |  |
| **Sex** | 10,912 |  |  | 100.0 | 2,327 |  |  | 100.0 |  |  |
| Female | 6,978 |  |  | 63.4 | 1,481 |  |  | 63.6 | 17.5 | 0.78 |
| Male | 3,934 |  |  | 36.1 | 846 |  |  | 36.4 | 17.7 |  |
| **Age** | 10,912 |  |  | 100.0 | 2,327 |  |  | 100.0 |  |  |
| 20-39 years | 1,015 |  |  | 9.3 | 118 |  |  | 5.1 | 10.4 | p <0.001 |
| 40-49 years | 1,109 |  |  | 10.2 | 177 |  |  | 7.6 | 13.8 |  |
| 50-59 years | 1,700 |  |  | 15.6 | 367 |  |  | 15.8 | 17.8 |  |
| 60-69 years | 4,737 |  |  | 43.4 | 1,179 |  |  | 50.6 | 19.9 |  |
| 70 yeas or more | 2,351 |  |  | 21.5 | 486 |  |  | 20.9 | 17.1 |  |
| **Education Attainment** | 10,912 |  |  | 100.0 | 2,327 |  |  | 100.0 |  |  |
| 9 years or less | 1,741 |  |  | 16.0 | 252 |  |  | 10.8 | 12.6 | p <0.001 |
| 10-12 years | 5,498 |  |  | 50.4 | 1,292 |  |  | 55.5 | 19.0 |  |
| 13 year or more | 3,518 |  |  | 32.2 | 739 |  |  | 31.8 | 17.4 |  |
| Others | 67 |  |  | 0.6 | 27 |  |  | 1.2 | 28.7 |  |
| Missing | 88 |  |  | 0.8 | 17 |  |  | 0.7 | 16.2 |  |
| **Loss of family and/or close relative and/or close friends in the disaster** | 10,912 |  |  | 100.0 | 2,327 |  |  | 100.0 |  |  |
| No | 5,182 |  |  | 47.5 | 586 |  |  | 25.2 | 10.2 | p <0.001 |
| Yes | 5,615 |  |  | 51.5 | 1,245 |  |  | 53.5 | 18.2 |  |
| Missing | 115 |  |  | 1.0 | 496 |  |  | 21.3 | 81.2 |  |
| **Household size** | 10,912 |  |  | 100.0 | 2,327 |  |  | 100.0 |  |  |
| Alone | 764 |  |  | 7 | 171 |  |  | 7.4 | 18.3 | p <0.001 |
| 2 or 3 | 6,073 |  |  | 55.6 | 1,405 |  |  | 60.3 | 18.8 |  |
| 4 or more | 3,816 |  |  | 35.0 | 710 |  |  | 30.5 | 15.7 |  |
| Missing | 259 |  |  | 2.4 | 41 |  |  | 1.8 | 13.7 |  |
| **Employment status** | 10,912 |  |  | 100.0 | 2,327 |  |  | 100.0 |  |  |
| Not working | 4,792 |  |  | 43.9 | 1,376 |  |  | 59.1 | 22.3 | p <0.001 |
| Working | 5,951 |  |  | 54.5 | 930 |  |  | 40 | 13.5 |  |
| Missing | 169 |  |  | 1.6 | 21 |  |  | 0.9 | 11.1 |  |
| **Housing damage** | 10,912 |  |  | 100.0 | 2,327 |  |  | 100.0 |  |  |
| No damage | 2,471 |  |  | 22.6 | 476 |  |  | 20.5 | 16.2 | 0.02 |
| Some damage | 7,962 |  |  | 73.0 | 1,764 |  |  | 75.8 | 18.1 |  |
| Missing | 479 |  |  | 4.4 | 87 |  |  | 3.70 | 15.4 |  |
| **Moving after baseline survey within coastal areas** | 10,912 |  |  | 100.0 | 2,327 |  |  | 100.0 |  |  |
| No | - |  |  | - | 2,100 |  |  | 90.2 | - | - |
| Yes | - |  |  | - | 227 |  |  | 9.8 | - |  |
| **Sea visibility** | 10,912 |  |  | 100.0 | 2,327 |  |  | 100.0 |  |  |
| No | 5,741 |  |  | 52.6 | 1,180 |  |  | 50.7 | 17.1 | 0.10 |
| Yes | 5,171 |  |  | 47.4 | 1,147 |  |  | 49.3 | 18.2 |  |
| **Proximity: closest distance to ocean** | 10,912 | 3.70 (±3.33) | (3.63, 3.76) | 100.0 | 2,327 | 3.67 (±3.24) | (3.54, 3.80) | 100.0 |  | 0.70 |
| Near | 6,801 |  |  | 62.3 | 1,484 |  |  | 63.8 | 17.9 | 0.20 |
| Far | 4,111 |  |  | 37.7 | 843 |  |  | 36.2 | 17.0 |  |

**S2 Results of multilevel mixed models with missing data (Model 1 and Model2) and multiple imputation for missing data (Model 1 with MI and Model 2with MI in Table 2).**

Ref: Reference category; MI: multiple imputation.

*p<0.05, **p<0.01, ***p<0.001.

|  | **Model 1** | | **Model 2** | | **Model 1 by MI** | | | | **Model 2 by MI** | | | | |
| --- | --- | --- | --- | --- | --- | --- | --- | --- | --- | --- | --- | --- | --- |
|  | **(two-way interaction)** | | **(three-way interaction)** | | **(two-way interaction)** | | | | **(three-way interaction)** | | | | |
|  | **Coef.** | **95％ CI** | **Coef.** | **95％ CI** | **Coef.** | **95％ CI** | | | **Coef.** | **95％ CI** | | | |
| **Intercept** | **6.04***** | 5.25, 6.84 | **6.30***** | 5.48, 7.14 | **5.95***** | 5.16, 6.74 | | | **6.21***** | 5.39, 7.04 | | | |
| **Effects of environments** | | | | | | | | | | | | | |
| **Sea visibility** | | | | |  | | | | | | | | |
| Non-visible | Ref. | - | Ref. | - | Ref. | - | | | Ref. | - | | | |
| Visible | 0.16 | -0.49, 1.64 | -0.26 | -0.85, 0.32 | 0.11 | -0.23, 0.46 | | | -0.29 | -0.87, 0.30 | | | |
| **Proximity (mean of closest distance to the ocean = 3.67 km)** | | | | | | | | | | | | | |
| Near (3.67 km or less) | -0.03 | -0.41, 0.35 | -0.03 | -0.41, 0.35 | -0.06 | -0.45, 0.33 | | | -0.06 | -0.45, 0.33 | | | |
| Far (over 3.67 km) | Ref. | - | Ref. | - | Ref. | - | | | Ref. | - | | | |
| **Two-way interaction effects of environments** | | | | | | | | | | | | | |
| **Visibility × Survey period (Reference= baseline)** | 0.06 | -0.26, 0.38 | 0.29 | -0.26, 0.84 | 0.07 | -0.26, 0.39 | | | 0.28 | -0.27, 0.82 | | | |
| **Proximity** × **Survey period** | -0.31 | -0.64, 0.02 | -0.03 | -0.41, 0.35 | -0.31 | -0.64, 0.02 | | | -0.03 | -0.65, 0.02 | | | |
| **(Reference= baseline)** |  |  |  |  |  |  |  |  |  |  |  |  |  |
| **Household number× visibility (Reference= non-visible)** | | | | | | | | | | | | | |
| Alone × visible | - | - | **1.33*** | 0.06, 2.59 | - | - | | | **1.28*** | 0.02, 2.54 | | | |
| 2-3 ×visible | - | - | 0.3 | -0.15, 0.75 | - | - | | | 0.5 | -0.20, 1.20 | | | |
| 4 or more × visible | - | - | Ref. | - | - | - | | | Ref. |  |  | - |  |
| Missing ×visible | - | - | 0.41 | -1.68, 2.50 |  |  |  |  |  |  |  |  |  |
| **Two-way interaction effect of time** | | | | | | | | | | | | | |
| **Household number × Survey period (Reference= baseline)** | | | | | | | | | | | | | |
| Alone× secondary | - | - | **1.08*** | 0.21, 1.95 | - | - | | | **1.10*** | 0.24, 1.97 | | | |
| 2-3×secondary | - | - | 0.03 | -0.15, 0.75 | - | - | | | 0.31 | -0.13, 0.76 | | | |
| 4 or more × secondary | - | - | Ref. | - | - | - | | | Ref. |  |  | - |  |
| Missing ×secondary | - | - | 0.41 | -1.68, 2.50 |  |  |  |  |  |  |  |  |  |
| **Three-way interaction effects of visibility** | | | | | | | | | | | | | |
| **Household number × visibility × Survey period (Reference= baseline)** | | | | | | | | | | | | | |
| Alone × visible × secondary | - | - | **-1.43**** | -2.65, -0.20 | - | - | | | **-1.43**** | -2.65, -0.21 | | | |
| 2-3 × visible × secondary | - | - | -0.19 | -0.84, 0.46 | - | - | | | -0.19 | -0.84, 0.46 | | | |
| 4 or more × visible × secondary | - | - | Ref. | - | - | - | | | Ref. |  |  | - |  |
| Missing × visible × secondary | - | - | -0.7 | -3.19, 1.78 |  |  |  |  |  |  |  |  |  |
| **Control variables** | | | | | | | | | | | | | |
| **Sex** | | | | | | | | | | | | | |
| Female | Ref. | - | Ref. | - | Ref. | - | | | Ref. | - | | | |
| Male | **0.54***** | -0.84, -0.24 | **0.55***** | -0.85, -0.25 | **-0.54***** | -0.84, -0.24 | | | **0.55***** | -0.85, -0.25 | | | |
| **Age** | | | | | | | | | | | | | |
| 20-39 years | 0.37 | -0.32, 1.06 | 0.35 | -0.34, 1.05 | 0.37 | -0.32, 1.06 | | | 0.35 | -0.35, 1.04 | | | |
| 40-49 years | Ref. | - | Ref. | - | Ref. | - | | | Ref. | - | | | |
| 50-59 years | -0.49 | -0.99, 0.02 | -0.47 | -0.97, 0.04 | -0.48 | -0.98, 0.03 | | | -0.46 | -0.96, 0.05 | | | |
| 60-69 years | **1.87***** | -2.37, -1.37 | **0.81***** | -2.35, -1.35 | **-1.85***** | -2.35, -1.34 | | | **1.83***** | -2.34, -1.33 | | | |
| ≤70 years | **2.07***** | -2.61, -1.52 | **2.26***** | -2.60, -1.51 | **-2.06***** | -2.61, -1.51 | | | **2.05***** | -2.60, -1.51 | | | |
| **Educational attainment** | | | | | | | | | | | | | |
| 9 years and less | Ref. | - | Ref. | - | Ref. | - | | | Ref. | - | | | |
| 10- 12 years | **-0.58*** | -1.05, -0.12 | **-0.57*** | -1.04, -0.10 | **-0.55*** | -1.02, -0.09 | | | **-0.54*** | -1.01, -0.08 | | | |
| 13 years and more | **-0.66**** | -1.17, -0.16 | **-0.66**** | -1.16, -0.16 | **-0.63*** | -1.13, -0.13 | | | **-0.63*** | -1.13, -0.12 | | | |
| Others | -0.86 | -2.22, 0.51 | -0.81 | -2.17, 0.56 | -0.82 | -2.20, 0.57 | | | -0.77 | -2.15, 0.61 | | | |
| Missing | 0.58 | -1.19, 2.35 | 0.6 | -1.17, 2.38 |  |  |  |  |  |  |  |  |  |
| **Loss of family and/or close relative and/or close friends in the disaster** | | | | | | | | | | | | | |
| No | Ref. | - | Ref. | - | Ref. | - | | | Ref. | - | | | |
| Yes | **0.65***** | 0.30, 1.01 | **0.64***** | 0.29, 1.00 | **0.64***** | 0.26, 1.04 | | | **0.64***** | 0.26, 1.03 | | | |
| Missing | 0.13 | -0.31, 0.56 | 0.13 | -0.31, 0.56 |  |  |  |  |  |  |  |  |  |
| **Household size (people)** | | | | | | | | | | | | | |
| Alone | **1.06***** | 0.49, 1.64 | 0.22 | -0.69, 1.13 | **1.07***** | 0.49, 1.64 | | | 0.24 | -0.67, 1.15 | | | |
| 2 or 3 | 0.18 | -0.15, 0.51 | -0.18 | -0.67, 0.31 | 0.2 | -0.15, 0.51 | | | -0.16 | -0.66, 0.33 | | | |
| 4 or more | Ref. | - | Ref. | - | Ref. | - | | | Ref. | - | | | |
| Missing | -0.3 | -1.39, 0.79 | -0.39 | -2.69, 1.92 |  |  |  |  |  |  |  |  |  |
| **Employment status** | | | | | | | | | | | | | |
| Not working | Ref. | - | Ref. | - | Ref. | - | | | Ref. | - | | | |
| Working | 0.18 | -0.12, 0.48 | 0.18 | -0.12, 0.48 | 0.19 | -0.11, 0.49 | | | 0.19 | -0.11, 0.49 | | | |
| Missing | -0.37 | -1.88, 1.14 | -0.36 | -1.87, 1.14 |  |  |  |  |  |  |  |  |  |
| **Housing damage** | | | | | | | | | | | | | |
| No damage | Ref. | - | Ref. | - | Ref. | - | | | Ref. | - | | | |
| Some damage | 0.18 | -0.17, 0.54 | 0.18 | -0.17, 0.54 | 0.19 | -0.17, 0.55 | | | 0.19 | -0.17, 0.54 | | | |
| Missing | -0.18 | -1.01, 0.65 | -0.19 | -1.02, 0.64 |  |  |  |  |  |  |  |  |  |
| **Moving after the baseline survey** | | | | | | | | | | | | | |
| No | Ref. | - | Ref. | - | Ref. | - | | | Ref. | - | | | |
| Yes | -0.22 | -0.71, 0.26 | -0.22 | -0.71, 0.27 | -0.22 | -0.71, 0.27 | | | -0.22 | -0.70, 0.27 | | | |
| **Survey period** | | | | | | | | | | | | | |
| Baseline | Ref. | - | Ref. | - | Ref. | - | | | Ref. | - | | | |
| Secondary | **1.32***** | -1.58, -1.06 | **1.58***** | -1.96, -1.19 | **-1.32***** | -1.58, -1.06 | | | **1.58***** | -1.97, -1.19 | | | |
| **Random effects** | | | | | | | | | | | | | |
| Individual-level | 8.90 | 8.19, 9.67 | 8.9 | 8.20, 9.67 | 2.98 | 2.86, 3.11 | | | 2.98 | 8.20, 9.67 | | | |
| School district-level | 0.03 | 0.00, 1.44 | 0.04 | 0.00, 1.32 | 0.23 | 0.06, 0.83 | | | 0.23 | 0.06, 0.82 | | | |
| **Residual variance** | 6.19 | 5.83, 6.57 | 6.17 | 5.81, 6.55 | 2.49 | 2.41, 2.56 | | | 2.48 | 2.41, 2.56 | | | |

**S3 Results of crude models (univariate multilevel mixed models) using each independent variable to predict K6 score with multiple imputation for missing data.**

***p<0.001, **p<0.01, *p<0.05

" MI: Multiple Imputation, CI: Confidence Interval.”

|  | **Crude model (Baseline survey)** | | **Crude model (Secondary survey)** | |
| --- | --- | --- | --- | --- |
|  | **Coef·** | **95％ CI** | **Coef·** | **95％ CI** |
| **Sea exposure** |  |  |  |  |
| **Sea visibility** | | | | |
| No (=0) | Ref. |  | Ref. |  |
| Yes (=1) | 0.28 | [-0.13, 0.69] | 0.12 | [-0.18, 0.42] |
| **Closest distance to the ocean (mean = 3.68 km)** | | | | |
| < mean | Ref. |  | Ref. |  |
| mean ≤ | -0.25 | [-0.75, 0.25] | 0.15 | [-0.21, 0.51] |
| **Basic characteristics** | |  |  |  |
|  |  |  |  |  |
| Female | Ref. |  | Ref. |  |
| Male | **-1.10***** | [-1.47, -0.72] | **-0.57***** | [-0.85, -0.29] |
| **Age** | | | | |
| 20-39 years | 0.11 | [-0.92, 1.14] | 0.11 | [-0.81, 10.2] |
| 40-49 years | Ref. |  | Ref. |  |
| 50-59 years | -0.97 | [-0.89, 0.69] | -0.52 | [-1.19, 0.16] |
| 60-69 years | **-1.98***** | [-2.68, -1.28] | **-1.46***** | [-2.04, -0.86] |
| ≤70 years | **-2.50***** | [-3.26, -1.74] | **-1.59***** | [-2.19, -0.99] |
| **Educational attainment** | | | | |
| 9 years and less | Ref. |  | Ref. |  |
| 10- 12 years | -0.24 | [-0.85, 0.37] | -0.20 | [-0.65, 0.26] |
| 13 years and more | -0.15 | [-0.80, 0.50] | -0.03 | [-0.50, 0.45] |
| Others | -1.79 | [-3.59, 0.01] | 0.11 | [-1.20, 1.43] |
| **Loss of family and/or close relative and/or close friends in the disaster** | | | | |
| No | Ref. |  | Ref. |  |
| Yes | **0.79**** | [0.30, 1.28] | 0.28 | [-0.06, 0.63] |
| **Household size (people)** | | | | |
| Alone | 0.37 | [-0.38, 1.11] | **0.71*** | [0.15, 1.27] |
| 2 or 3 | **-0.48*** | [-0.88, -0.07] | -0.20 | [-0.50, 0.11] |
| 4 or more | Ref. |  | Ref. |  |
| **Employment status** | | | | |
| Not working | Ref. |  | Ref. |  |
| Working | **-0.47*** | [-0.84, -0.10] | 0.02 | [-0.26, 0.30] |
| **Housing damage** | | | | |
| No damage | Ref. |  | Ref. |  |
| Some damage | 0.25 | [-0.22, 0.72] | 0.08 | [-0.26, 0.42] |
| **Moving after baseline survey** | | | | |
| No | - |  | Ref. |  |
| Yes | - |  | **0.88***** | [0.42, 1.34] |

**S4 Results of Predictive Margins of K6 change (dy/dx) by the interaction term of sea visibility and household size in Fig 1.**

| Table (A). Average marginal effects of sea visibility for living alone samples: contrast of average marginal effects of time by visibility | | |
| --- | --- | --- |
| Sea view | dy/dx | 95%CI |
| Non-visible | -0.64 | [-1.43, 0.15] |
| Visible | -1.91*** | [-2.69, -1.13] |
|  |  |  |
| Table (B). Test of within each factor of living alone: contrast of average marginal effects of time by proximity | | |
| Sea view | dy/dx | 95%CI |
| Non-visible | ref. |  |
| Visible | -1.27*** | [-2.38, -1.67] |

*** stands for the significance at the 0.1% level.

**stands for the significance at the 1% level.

*stands for the significance at the 5% level.

dy/dx is the discrete change of margins from the baseline to the follow-up.

P-value is obtained from Chi-square test of the difference in dy/dx between compared factors by using Delta-method.

**S5 Questionnaire items related to this study in Japanese (original) and English (translated).**

The following questions with English translations are extracted from the original questionnaire only as they relate to this study. The original questionnaire was designed in Japanese only, and this English translation has been made by the authors for the convenience of the readers of this article.

**(Japanese version)**

東北メディカル・メガバンク機構　調査票

健康と生活習慣に関するおたずね

| 氏名 | 電話番号 |
| --- | --- |
| 現住所 | |

**基本情報**

| ①　性別　a. 男　b.　女 |
| --- |
| ②　年齢　満（　）歳 |
| ③　最後に卒業された学校（在学中の方は現在のもの）  a. 小・中学校　b.高校　c.専門学校　d. 短大・高専　e.大学　f.大学院  g.その他（　　） |
| ④震災時にお住まいのご自宅の被害情報について、当てはまるもの1つに〇をつけてください。  a.全壊（全壊流失）b. 大規模半壊　c. 半壊　d. 一部損壊　e. 損壊なし  f. 被災地に居住していない |

**ストレスについておたずねします（K6）。**

①心の元気さについておたずねします。（過去1か月間についてお答えください）

次のそれぞれの質問について当てはまるものに〇をつけてください。

|  | いつも | たいてい | ときどき | 少しだけ | 全くない |
| --- | --- | --- | --- | --- | --- |
| 神経過敏に感じましたか | a | b | c | d | e |
| 絶望的だと感じましたか | a | b | c | d | e |
| そわそわ、落ち着かなく感じましたか | a | b | c | d | e |
| 気分が沈み込んで、何が起こっても気が晴れないように感じましたか | a | b | c | d | e |
| 何をするにも骨折りだと感じましたか | a | b | c | d | e |
| 自分は価値のない人間だと感じましたか | a | b | c | d | e |

家族構成についておたずねします。

| ①あなたは、ご自身を含めて何人で暮らしていますか？　　（　）人 |
| --- |

お仕事の状況についておたずねします。

| 現在の仕事について、当てはまるもの１つに〇をつけてください。  a. 正社員　b. 自営・またはその手伝い　c. 派遣社員　d. パート・アルバイト・嘱託e. 休職中　f.　仕事をしていない（年齢受給者、主婦、学生、無職含む） |
| --- |

東日本大震災での被災と喪失の体験についておたずねします

| あなたの親しい方の中で震災の影響によって亡くなった方、もしくは行方不明の方はいらっしゃいますか？  a. はい　b.　いいえ |
| --- |

----------------------------------------------------------------------------------------------------------

**(English translation)**

Tohoku Medical Megabank Organization

Community-Based Cohort Study on Health and Lifestyles

| Name: | Tel: |
| --- | --- |
| Address: | |

**Basic information**

| ①　Sex:　a. Male　b.　Female |
| --- |
| ②　Age:　（　）years old |
| ③　Your last school (or current one if you are still studying)  a. Elementary school / junior high school　b. High school　c. Vocational school　d. 2-year college or college of technology　e. University　f.　Graduate School　g. Others ( ) |
| ④ Please tick one answer that best describes the damage to your house at the time of the disaster.  a. Completely destroyed (including swept-away by water) b. Large-scale half-destroyed　c. Half-destroyed　d. Partially damaged　e. No damage　f. Lived outside the disaster affected areas |

**Question about your stress（K6）**

1. Question about your mental wellbeing (Please think about the past one month). Please tick the answer that best describes each of the following questions.

|  | All | Most | Some | A little | None |
| --- | --- | --- | --- | --- | --- |
| About how often did you feel nervous? | a | b | c | d | e |
| About how often did you feel hopeless? | a | b | c | d | e |
| About how often did you feel restless or fidgety? | a | b | c | d | e |
| About how often did you feel so depressed that nothing could cheer you up? | a | b | c | d | e |
| About how often did you feel that everything was an effort? | a | b | c | d | e |
| About how often did you feel worthless? | a | b | c | d | e |

Question about your family members

| 1. How many persons do you live with, including yourself? 　( ) |
| --- |

Question about your employment situation

| Please tick one answer that best describes your current employment situation.  a. Regular employee　b. Self-employed or assistant　c. Dispatched employee　d. Part-time employee / temporal employee / contract employee e. Leave of absence　f. Not working (includes Pensioner, Housewife, Student, Unemployed) |
| --- |

Question about your experience of suffering and loss in the Great East Japan Earthquake and Tsunami.

| Have any of your loved ones died or gone missing due to the disaster?  a. Yes　b. No |
| --- |
